# Treatment of Burns

**Published:** 1850-07

**Authors:** 


*Treatment of Burns.*—Perhaps there is no accident in the catalogue of surgery, wherein greater discrepancy of opinion exists regarding the best treatment, in the primary condition, than that of burns. One succeeds best by the antiphlogistic, another by the stimulating plan—while some contend for a medium between the two. It appears to us, that there can be but one principle upon which should be based the primary treatment of burns, and that is the vital one of inflammation. It is most emphatically inflammation, and of course attended with its results, either in a low or high degree, according as the parts are injured. A simple burn, or that considered of the first degree, wherein there is slight redness of the skin, will readily resolve itself, like unto all simple inflammations, without any, or the mildest and most simple treatment. But when an extensive surface is invaded, and the parts beneath are deeply involved, then the matter is entirely different, and requires a prompt and active treatment. The location of the lesion has something to do in our management and treatment of such injuries; for burns upon the head, face, neck, thorax and abdomen, produce greater constitutional symptoms, and are more dangerous to life, than the same extent of injured surface npon the extremities. The indication, then, required in our management, is, to lessen the pain, and make the inflammatory process as little destructive as possible; aud if it cannot be prevented, then, of course, to promote the separation of the dead parts from the living, in a manner best calculated for the comfort and safety of the patient. We have lately had an extensive burn under our care, in the case of a lady, who, while attempting to give what is called a *rum sweat* to another lady, had her own dress take fire from the burning alcohol, nearly all of which was consumed upon her body before any one could render her the least assistance. We were upon the spot within ten minutes after the accident, and found the patient in a very bad condition. Both arms, from the elbows to the top of the shoulder, were much burned; also the left side, up to the axilla, and on to the top of the thorax. There were many kind friends present; some recommending most of the popular quack remedies of the day, while others were for the usual domestic remedies, some of them quite rational. Linen cloths were dipped in alcohol and water, and the burn was dressed with them. They were not suffered to be removed for twenty hours, and during this time they were kept thoroughly saturated with the cold water and alcohol; at the commencement, equal parts of each, but subsequently two thirds of alcohol to one of water. The patient was relieved from pain upon the first application, and would have passed the time in a very comfortable manner, had she not suffered from the nervous excitement produced by the accident and the usual constitutional symptoms attendant upon such an extensive injury. The subsequent treatment has been to leave the parts undressed, or simply smear them over with gum water. On the sixth day (at the time of this notice) there is no appearance of sloughing, and the patient has rallied, and is very comfortable. This plan is recommended by Mr. Kentish; and the simplicity of the dressings, together with the comfort that is afforded the patient, should be sufficient inducements for every practitioner to make use of it without any prejudice.—*Boston Med. and Surg. Journal,* (*Editorial.*)

